# Honey Bees (*Apis mellifera*, L.) as Active Samplers of Airborne Particulate Matter

**DOI:** 10.1371/journal.pone.0132491

**Published:** 2015-07-06

**Authors:** Ilaria Negri, Christian Mavris, Gennaro Di Prisco, Emilio Caprio, Marco Pellecchia

**Affiliations:** 1 Koiné—Environmental Consulting S.n.c., Parma, Italy; 2 Department of Earth Sciences, Natural History Museum, London, United Kingdom; 3 Dipartimento di Agraria, Laboratorio di Entomologia E. Tremblay, Università degli Studi di Napoli Federico II, Portici (Napoli), Italy; San Diego, UNITED STATES

## Abstract

Honey bees (*Apis mellifera* L.) are bioindicators of environmental pollution levels. During their wide-ranging foraging activity, these hymenopterans are exposed to pollutants, thus becoming a useful tool to trace the environmental contaminants as heavy metals, pesticides, radionuclides and volatile organic compounds. In the present work we demonstrate that bees can also be used as active samplers of airborne particulate matter. Worker bees were collected from hives located in a polluted postmining area in South West Sardinia (Italy) that is also exposed to dust emissions from industrial plants. The area is included in an official list of sites of national interest for environmental remediation, and has been characterized for the effects of pollutants on the health of the resident population. The head, wings, hind legs and alimentary canal of the bees were investigated with Scanning Electron Microscopy coupled with X-ray spectroscopy (SEM-EDX). The analyses pointed to specific morphological and chemical features of the particulate, and resulted into the identification of three categories of particles: industry -, postmining -, and soil –derived. With the exception of the gut, all the analyzed body districts displayed inorganic particles, mostly concentrated in specific areas of the body (i.e. along the costal margin of the fore wings, the medial plane of the head, and the inner surface of the hind legs). The role of both past mining activities and the industrial activity close to the study area as sources of the particulate matter is also discussed. We conclude that honey bees are able to collect samples of the main airborne particles emitted from different sources, therefore could be an ideal tool for monitoring such a kind of pollutants.

## Introduction

Honey bees (*Apis mellifera* L.) are commonly used as bioindicators of the level of environmental contamination. During their wide-ranging foraging activity, these hymenopterans are exposed to pollutants present in the atmosphere, soil, vegetation, and water [1–3]. Depending on the type of environmental pollution, bee contamination may occur through adhesion of particles to the insect body hairs, inhalation of pollutants via spiracles of the tracheal system or ingestion of contaminated nectar, pollen and water. Contaminants are brought back to the hives and may also be found into the apiary products, such as honey and wax [4–6].

Among environmental contaminants found in honey bees and bee products, the most commonly studied are heavy metals, pesticides, radionuclides and Volatile Organic Compounds (VOCs) [1, 4, 6–8]. Despite the well-known role of honey bees in environmental monitoring, studies using these hymenopterans as active samplers of airborne particulate matter (PM) are completely lacking, even if the morphological description and the physico-chemical characterization of PM collected by the bees would provide accurate information on both the emission source(s) and the potential health hazards [9–11]. Indeed, this is a key point for developing adequate control strategies in order to reduce the impact of pollutants on both the environment and public health.

Studies on atmospheric pollutants include the vast field of airborne particulate matter. PM is broadly defined as a complex mixture of airborne chemical components which are commonly classified by particle size. They include ultra-fine particles (up to 0.1 μm in diameter), fine particles or PM1 (up to 1 μm), PM 2.5 (up to 2.5 μm), coarse fraction or PM 10 (up to 10 μm). The airborne particles ≤100 μm in diameter are collectively referred as total suspended particulate (TSP).

PM can be directly emitted as primary compounds or formed as secondary compounds by chemical transformation or condensation of gases such as SOx, NOx, VOCs and ammonia. Primary sources comprise both natural sources, such as windblown dust, volcanic eruptions, forest fires and sea spray, and anthropogenic activities. The latter represent a broader domain, ranging from agricultural operations to industrial processes, mining and postmining activities, combustion of wood and fossil fuels, incineration of wastes and motor traffic (vehicles, aircrafts, ships, trains), etc. [12–15].

Over the years, several human diseases have been linked to PM exposure, which may be responsible for short-term, long-term and cumulative health effects [16–20]. Neonatal premature mortality, morbidity, cardiovascular and cardiopulmonary diseases, asthma and lung cancer are among the more frequent effects observed in patients exposed to airborne particles [16, 18, 19]. Toxicological researches have shown that, at a cellular level, PM may induce cytotoxicity, neurotoxicity, mutagenicity, stimulation of pro-inflammatory factors, and even epigenetic alterations of the DNA with consequences on gene expression [19, 21, 22].

Moreover, the size of the particles and their surface area determine the potential to elicit the adverse biological effects. Ultra-fine particles are of much concern, as they can penetrate deeper into the airways of the respiratory tract, enter blood circulation, and then distribute to most organs, including the brain [19, 21].

The aim of this work was to investigate the role of honey bees as active samplers of PM.

The study was carried out in a post-mining area of Sulcis-Iglesiente, in the municipality of Iglesias (Carbonia-Iglesias province, Sardinia, Italy). Sulcis-Iglesiente is included in an official list of sites of national interest for environmental remediation and has been characterized for the effects of pollutants (mostly metals and metalloids deriving from past mining activities) on the health of the resident population [23, 24]. The PM collected by the worker bees on the body was analyzed using a Scanning Electron Microscope (SEM) coupled with X-ray spectroscopy (EDX). The dissected alimentary canal of the hymenopterans was also investigated to detect inorganic particles potentially ingested during feeding.

## Materials and Methods

### Study area

**Fig 1:**
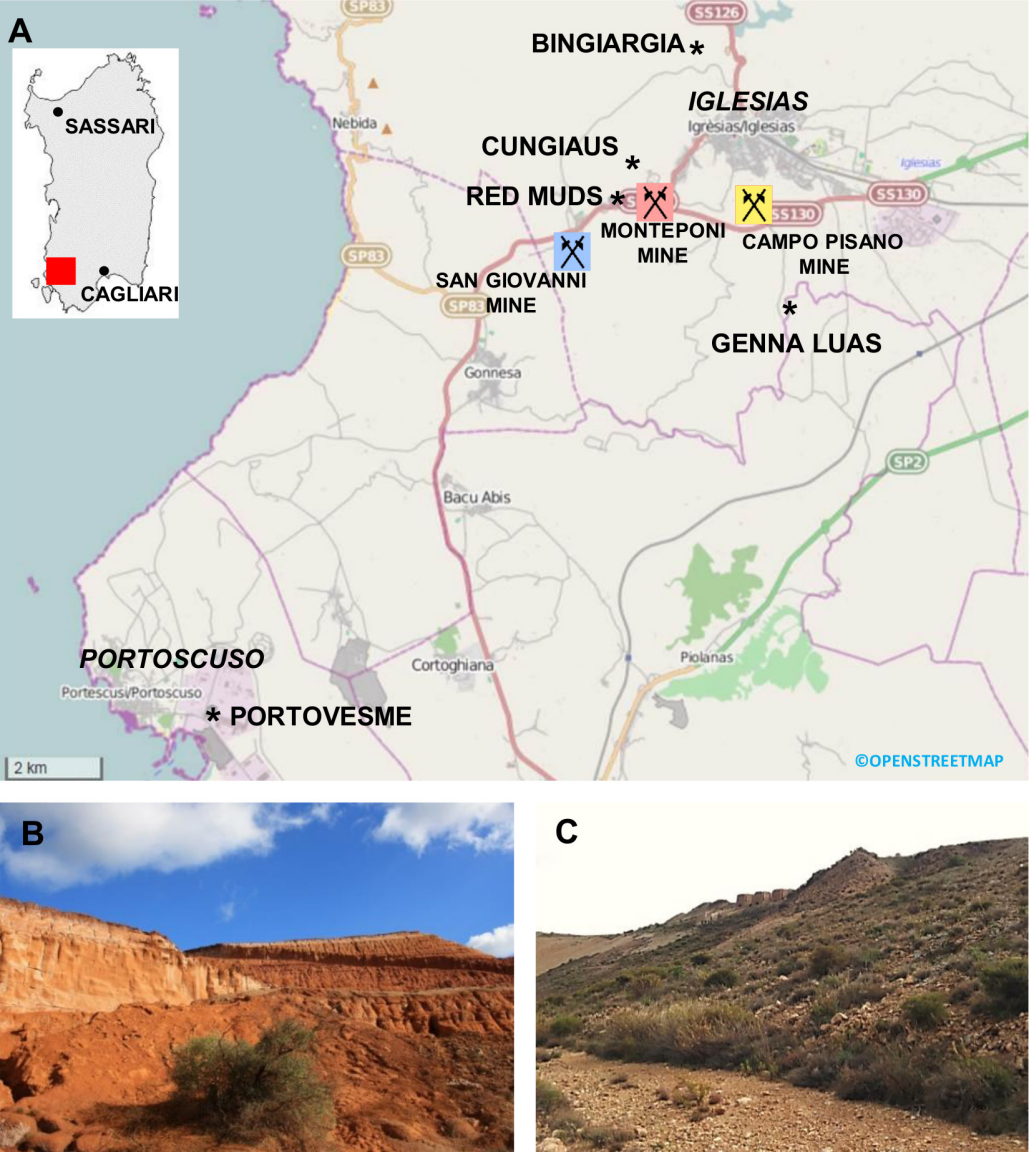
(A) Study area; (B) view of the Red Muds, and (C) the mine dumps of Cungiaus.

The town of Iglesias is located in South West Sardinia, about 50 km West of Cagliari (Fig 1A). Its surroundings are known for the baryte and Pb-Zn ore deposits, extensively exploited during the Nineteenth Century and until recent times through dozens of mines. Among them, the Pb-Zn mines of Monteponi, Campo Pisano and San Giovanni are certainly the most famous (Fig 1A) [25] and, together with about 40 mines—spread out over an area of 150 km^2^ –they exploited the deposits of the so called “Metalliferous Ring” [25, 26].

As a result of those intense mining operations, several million metric tons of ore material were extracted, leaving to broad daylight extensive tailings. This is especially the case of the Monteponi mining complex, located immediately West of Iglesias. In particular, the treatment of the oxidized Zn-ores (calamines) was carried out in an electrolytic plant, which involved fine grinding (typically <40 μm grains) of calamines and then their treatment with sulphuric acid, FeSO_4_ and MnO_2_ for the enrichment of the Zn concentrate. The wastes from this process were deposited downstream of the industrial complex and nowadays constitute the hill of the Red Muds (“Fanghi Rossi” in Italian; RM in this work) (Fig 1B). The hill—which is subjected to preservation regulations as an industrial archaeology site—mainly contains iron oxy-hydroxides associated with Zn-silicates and carbonates, gypsum, and toxic elements such as Cd, Pb, As, Hg, Mn, and Ba [26–27]. In total, the RM cover an area of about 15 ha with a volume of 500,000 m^3^, occupying a major part of the lowest, West—South West flank of the mined hill of Monteponi. At the present state, the RM sediments are only contained by wooden bulkheads. The steep slopes, along with the fine grain size of the material, promote intense weathering of the postmining materials. During rain events, runoff transfers the sediment load to the surrounding areas via the San Giorgio creek, which lies immediately downhill [26–27].

North of Monteponi, the open pit of Cungiaus is the largest of Sardinia, covering more than 10 ha of surface. Past mining activities exploited an extensive mass (about one million m^3^) of calamines since 1869 (the year of discovery) to the first half of the Twentieth Century [25, 28].

Besides the past intensive mining activity, Sulcis-Iglesiente is also exposed to emissions by industrial plants, mostly located along the South West seashore (industrial district of Portovesme). Portovesme is approximately 8 Km South West of Iglesias, in the municipality of Portoscuso (Fig 1A). The main industrial plants include different units: a sector for the production of alumina from bauxite and the production of aluminum by electrolysis of alumina (currently in standby); electric power stations, composed by a coal-powered generation plant and an oil-powered plant; and a Pb-Zn smelter that uses steelwork dusts for Zn extraction. This type of smelter is known to produce post-processing atmospheric fall-out impacting on the immediate surroundings [29].

### Hives and honey bees

Eleven hives were located in Bingiargia (39°19’31”N–08°31’07”E), a Mediterranean scrub area just outside the town of Iglesias (Fig 1A). At the beginning of November 2013, twenty worker bees were sampled alive with a butterfly net, while returning to their hives. The climate was characterized by warm and sunny weather. Honey bees were collected at 11 a.m. with a temperature of 23°C. The bees were immediately put in soda glass capped vials (Chromacol Limited), stored on ice in order to keep them inactive, and quickly brought to lab for sample preparation.

After a few hours at -20°C, heads, wings and hind legs were cut under a stereoscope with scalpels and ophthalmological scissors, and mounted onto SEM stubs using double adhesive carbon tape.

We excluded from the analyses the other two pairs of legs because preliminary SEM observations demonstrated that PM almost predominantly concentrates on the hind legs, in particular on their inner surface, following the antero-posterior “handling” of the pollen [30].

In order to analyze the gut content and the intestinal wall, the remaining body (thorax and abdomen) was put in sterile saline solution and the alimentary canal dissected. After dehydration through 70%, 80%, 90% (one passage for 20min) and 100% (two passages for 20min) ethanol series, the entire alimentary canal was mounted onto SEM stubs. Later, honey stomach, ventriculum and rectum were longitudinally cut, gently opened and air-dried, in order to preserve the gut content.

A few days later, ten worker-bees were collected (as control samples) in a rural area 10 km South of Parma (Northern Italy), near the bed of Parma creek and close to the foothills of the Apennine Mountains (44°41’24.7”N–10°20’9.9”E). Worker bees were sampled at 1 p.m. with a temperature of 19°C. The weather was partly cloudy. This control site (CS) was far from any known emitting sources of PM (i.e. vehicular traffic, incinerators, cement plants, industries, etc.). The hives were placed along the creek floodplain, and the surrounding hills consisted mainly of sandstones, marls and calcarenites, with noticeable clayey layers [31].

The preparation technique applied to the control bees was identical to the Sardinian ones.

All honey bees used in this study were collected in the presence of the beekeepers and with the permission of the owners of the private land were the hives were located.

No endangered or protected species are involved in this research.

### Sediment samples

In order to identify the potential source(s) of the PM detected on the Sardinian honey bees, specific candidate sites were selected: Bingiargia (BNG; private land sampled with the owner permission), Monteponi Red Muds (RM; 39°17’52.8”N–08°30’25.2”E) and Cungiaus (CUN; 39°18’29”N–08°30’27”E) (Fig 1). The authors are not aware of any restriction regarding the sampling of soil sediments in the RM and CUN sites.

Main choice criterion of the sites was the exposure to wind uptake. BNG samples were collected up to 10 m from the hives. While BNG site undoubtedly fell within the foraging range of the apiary, CUN and RM possibly did not because they were quite distant from the apiary, i.e. about 3 and 3.5 Km far, respectively, and field studies have demonstrated that in autumn the average foraging distance achieved by the honey bees is less than 1.5 Km, reaching a maximum in the summer of about 2.2 Km [32]. In addition, foraging bees were exclusively collecting honeydew from holm oaks (*Quercus ilex*), which were absent in both sites.

With the aim of making a direct comparison between the mineral particles detected on the honey bees and the wind-available fraction of the soil, topsoil/exposed sediment samples were collected and then investigated with the same analytical technique (SEM-EDX).

In each candidate site, 3 to 5 samples were collected: shallow pits were excavated (down to max 5 cm) and up to 0.5 kg of material was recovered per pit. Samples were air-dried and an aliquot (about 1 g) was mixed with all other samples from the same site in order to obtain a representative group sample. The obtained mixture was then poured and mounted onto SEM stubs using double adhesive carbon tape. Despite the obvious grain size heterogeneity, only particles <100 μm in diameter (i.e. the fraction which can account for the TSP) were investigated for this study. Five replicates were analyzed for each soil sample.

The CS soil composition was not measured, but derived by literature [31, 33]. Soils of the area are mostly developed over fluvial sediments of various grain sizes, ranging from clays to conglomerate of sedimentary origin (Holocene–Upper Pleistocene, and older) [31, 33].

### SEM-EDX analysis

SEM-EDX measurements were carried out on both the dissected portions (wings, head, hind legs, alimentary canal) of the honey bees and sediment samples. No coatings or other treatments were applied. Charging artifacts were largely suppressed using the low vacuum mode (100 Pa water vapor) at room temperature in a SEM FEI Quanta 200 FEG, equipped with an Ametek-EDAX ApolloX analytical system. Secondary Electrons (SE) and BackScattered Electrons (BSE) images, as well as EDX point analyses, were acquired in alternating sequence at the same conditions of 20 kV with a nominal beam current of about 1 nA, in order to provide the chemical composition, morphology, surface characteristics and size of the particles.

Each EDX spectrum was then interpreted according to a mineralogical point of view, also taking into account both the simultaneous presence of multiple phases and mineral content of the surrounding geological formations.

## Results

### Honey bees: wing and body surface

**Fig 2:**
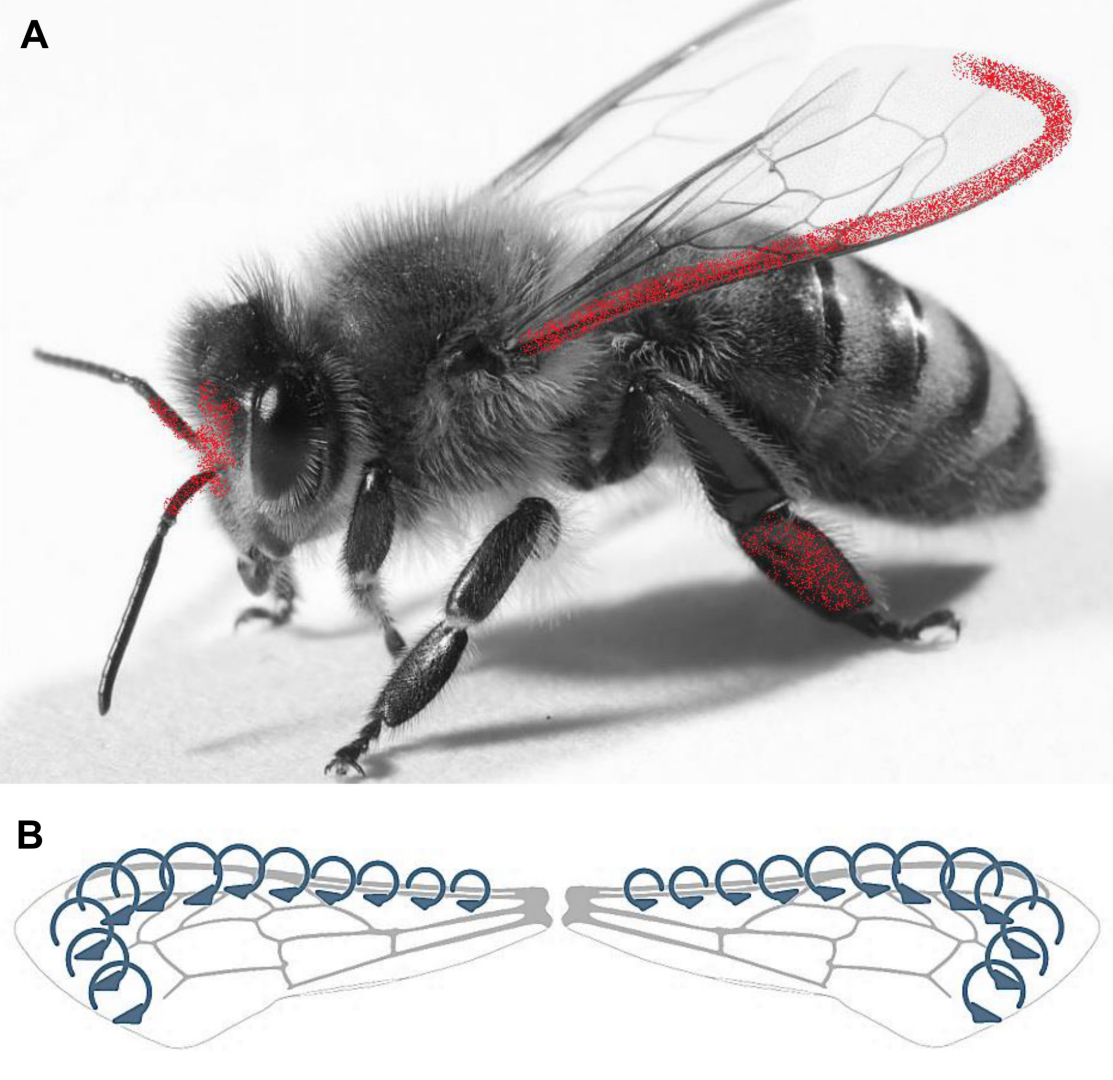
(A) Airborne PM (red) on the honey bees is mostly concentrated along the costal margin of the fore wings, the medial plane of the head, and the inner surface of the hind legs. (B) The Leading Edge Vortex (LEV) formed at the leading edge of the fore wings during the insect flight.

A detailed investigation of all the worker bees sampled in Sardinia revealed high contamination due to thousands of inorganic particles on the external body districts (i.e. head, hind legs and wings), mostly concentrated in specific areas (Fig 2A).

**Fig 3:**
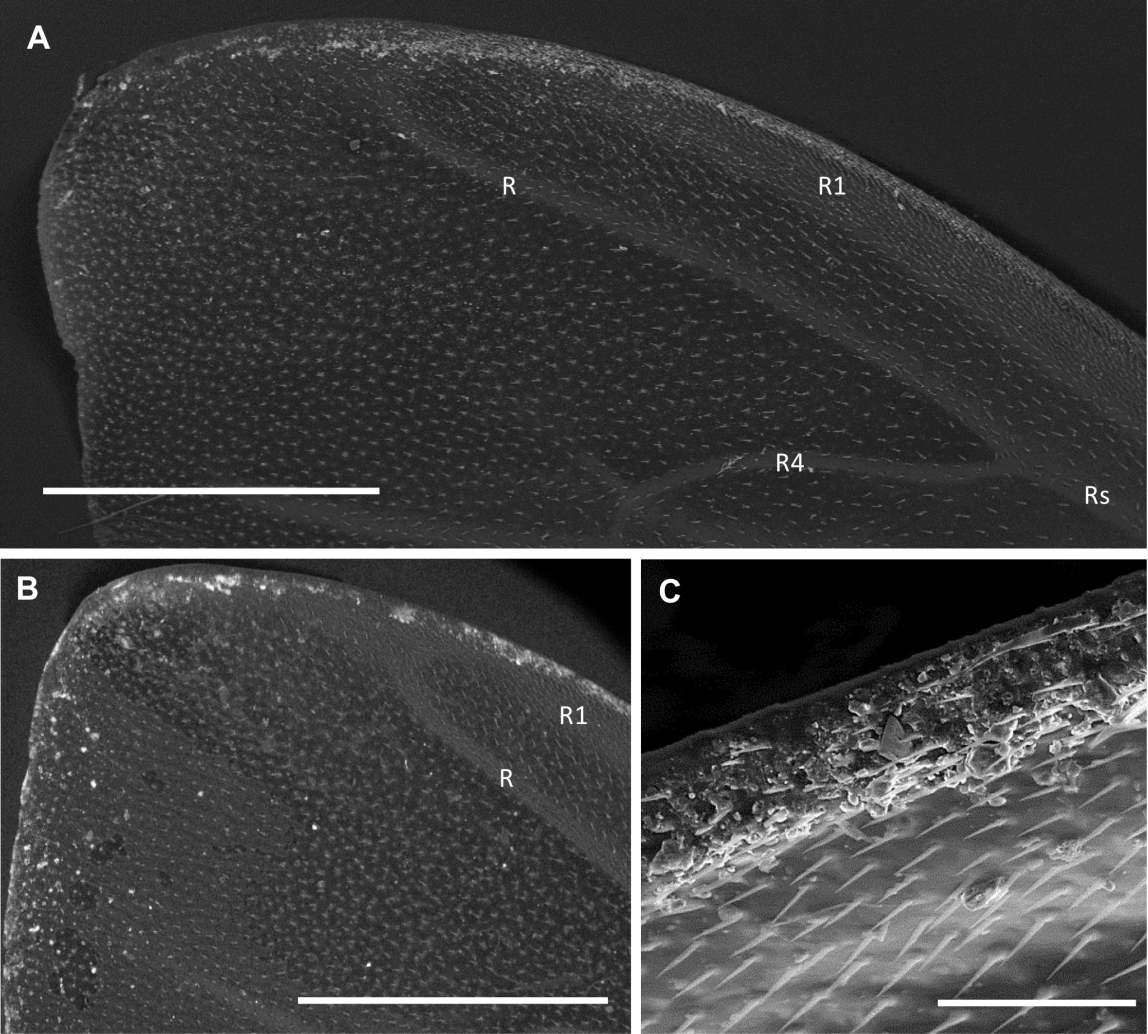
SEM images of the fore wings partially covered with PM. (A, B) Fore wings of Sardinian worker bees displaying PM (bright spots) most concentrated along the costal margin lining the first branch of the radial vein and the apex. BSE images. Bar = 1 mm. (C) A detail of particles gathered along the first branch of the radial vein. SE image. Bar = 100 μm. R = radial vein; R1 = first branch of the radial vein; Rs = second branch or radial sector; R4 = fourth branch.

In all specimens, a large amount of particles was observed on the fore wings (upper surface), along the costal margin lining the first branch of the radial vein and the apex (Figs 2A and 3).

Fewer particles were dispersed on the remaining sector of the fore wings (Fig 3A and 3B) and on the hind wings (S1 Fig).

**Fig 4:**
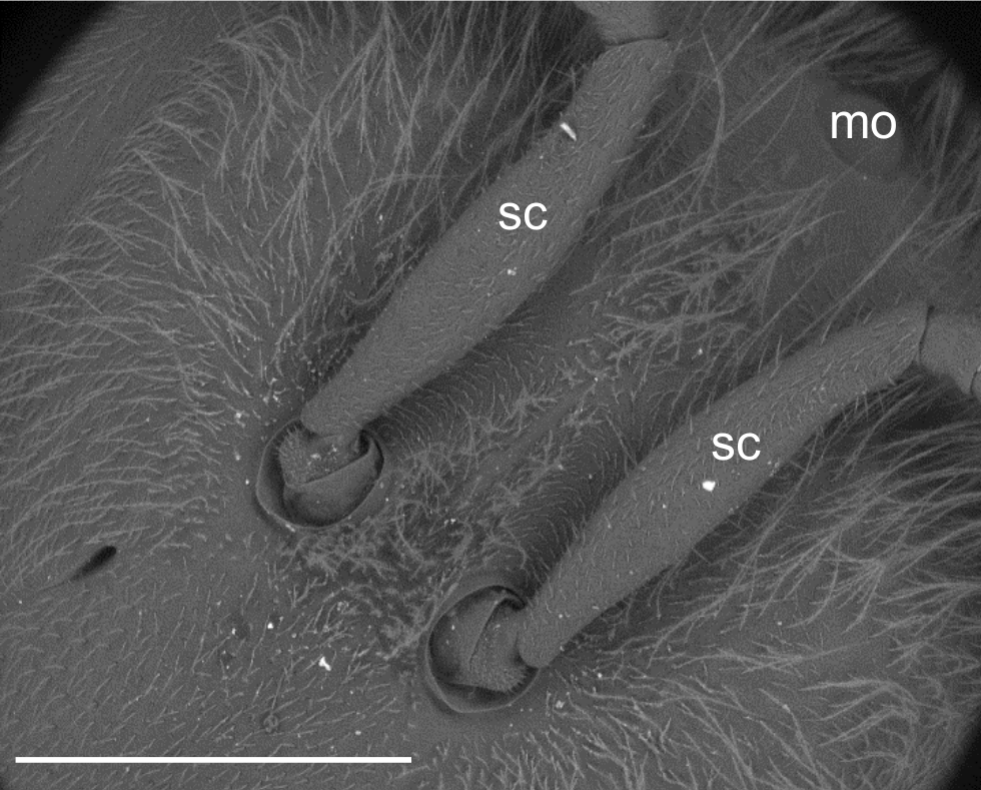
Honey bee head displaying PM (bright spots) almost exclusively along the medial plane, nearly between the median ocellus (mo) and the antennae, including the scapus (sc). BSE image. Bar = 1 mm.

Heads showed particles almost exclusively along the medial plane, in a narrow area nearly between the bases of the antennae and the median ocellus (Figs 2A and 4); PM was also observed on the scape of each antenna (Fig 4).

**Fig 5:**
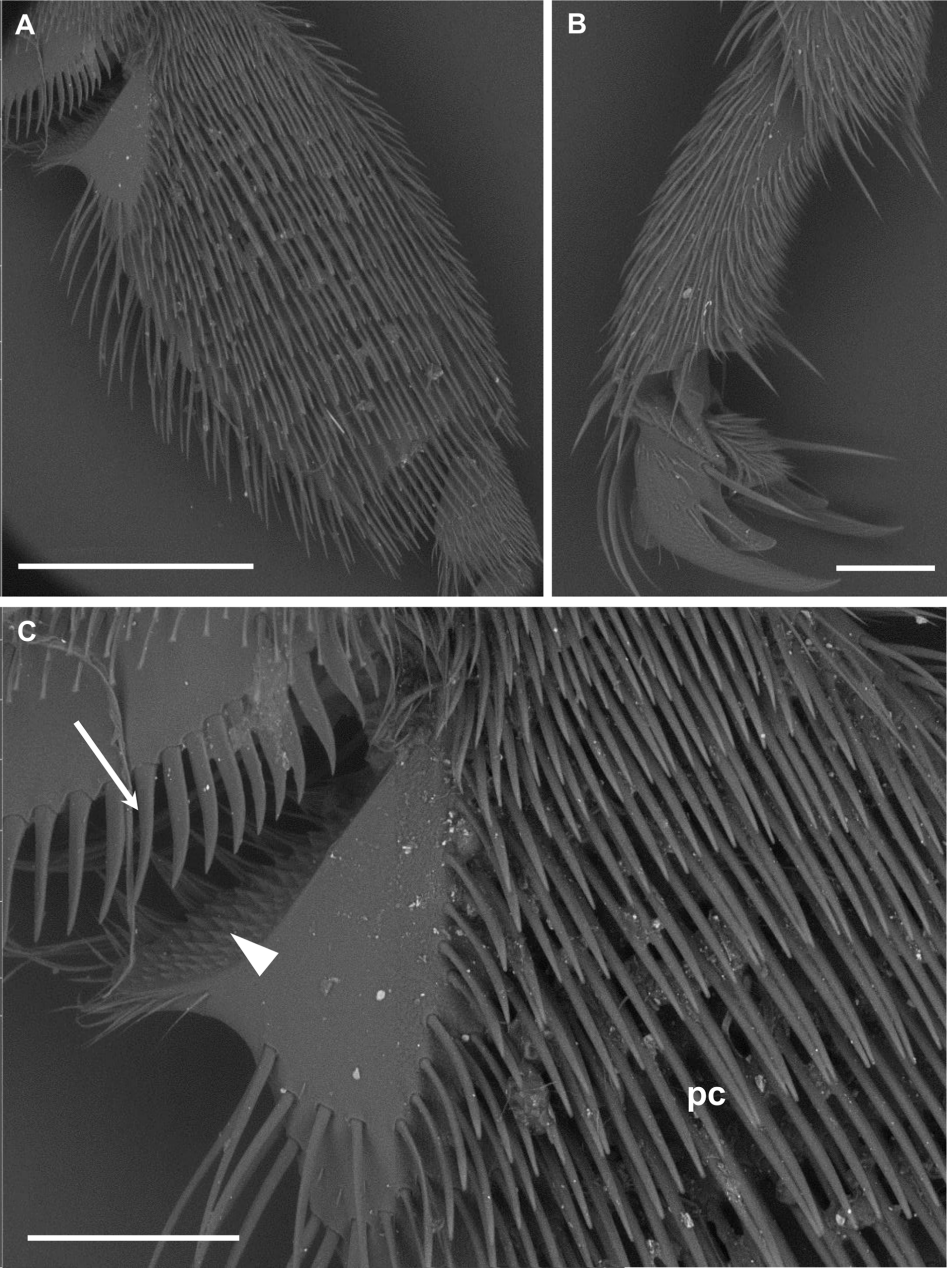
BSE images of PM (bright spots) on the hind legs. (A) Metatarsus. Bar = 1 mm. (B) Distal tarsal segments. Bar = 150 μm. (C) Detail of the structures involved in the grooming behavior and pollen collection. The pecten spines (arrow) and the pyramidal spines of the auricle (arrowhead) convey and pack the pollen into the pollen basket located on the outer surface of the leg. The pollen comb (pc), composed by transverse rows of stiff spines, brush off pollen from the lateral surface of the body and collect wax scales from the abdomen. Bar = 300 μm.

On the third pairs of legs, the coverage of inorganic particles was always rather diffused along the most distal segments of the inner surface, and involved the structures dedicated to the body grooming, pollen collection, and wax handling (e.g. the pecten on the lower end of the tibia and the pollen comb on the metatarsus) (Figs 2A and 5).

**Fig 6:**
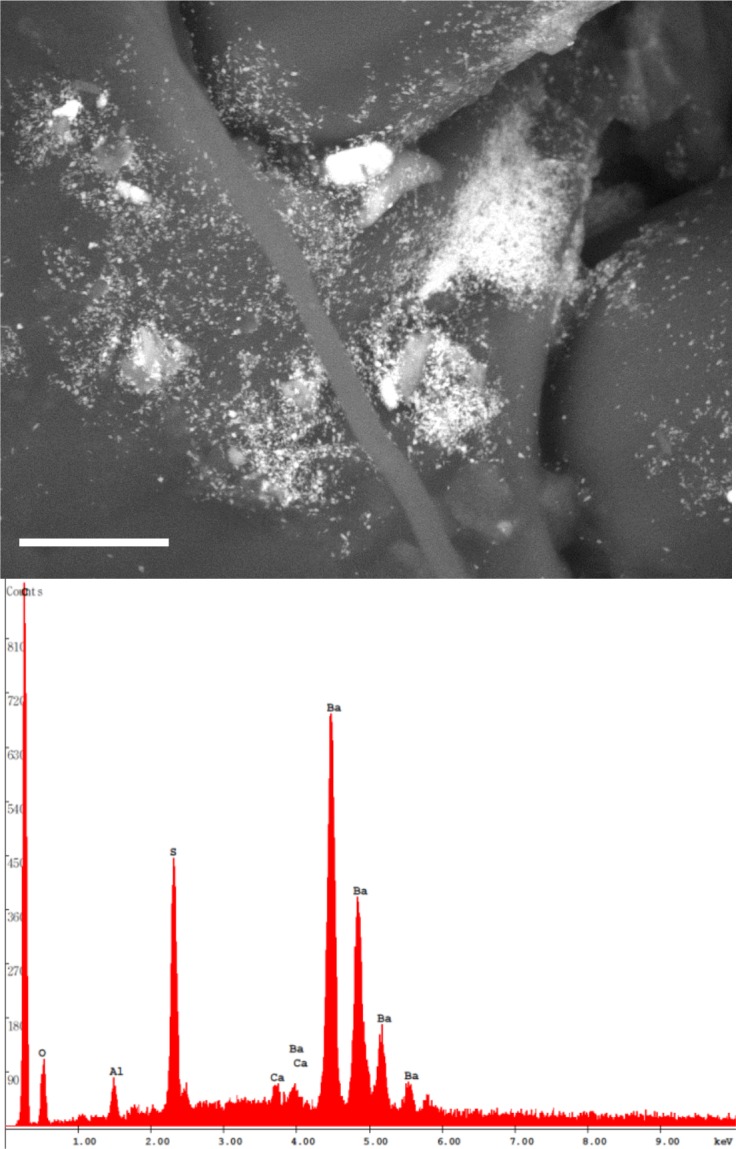
Fine and ultra-fine particles of baryte evenly spread across the honey bee wing. BSE image. Bar = 10 μm.

Particle size was rather various and ranged from a few nm to 50 μm. Where present, the ultra-fine and fine particles were uniformly spread across the scanned surfaces (Fig 6); EDX analysis revealed that they were always fragments of baryte.

**Fig 7:**
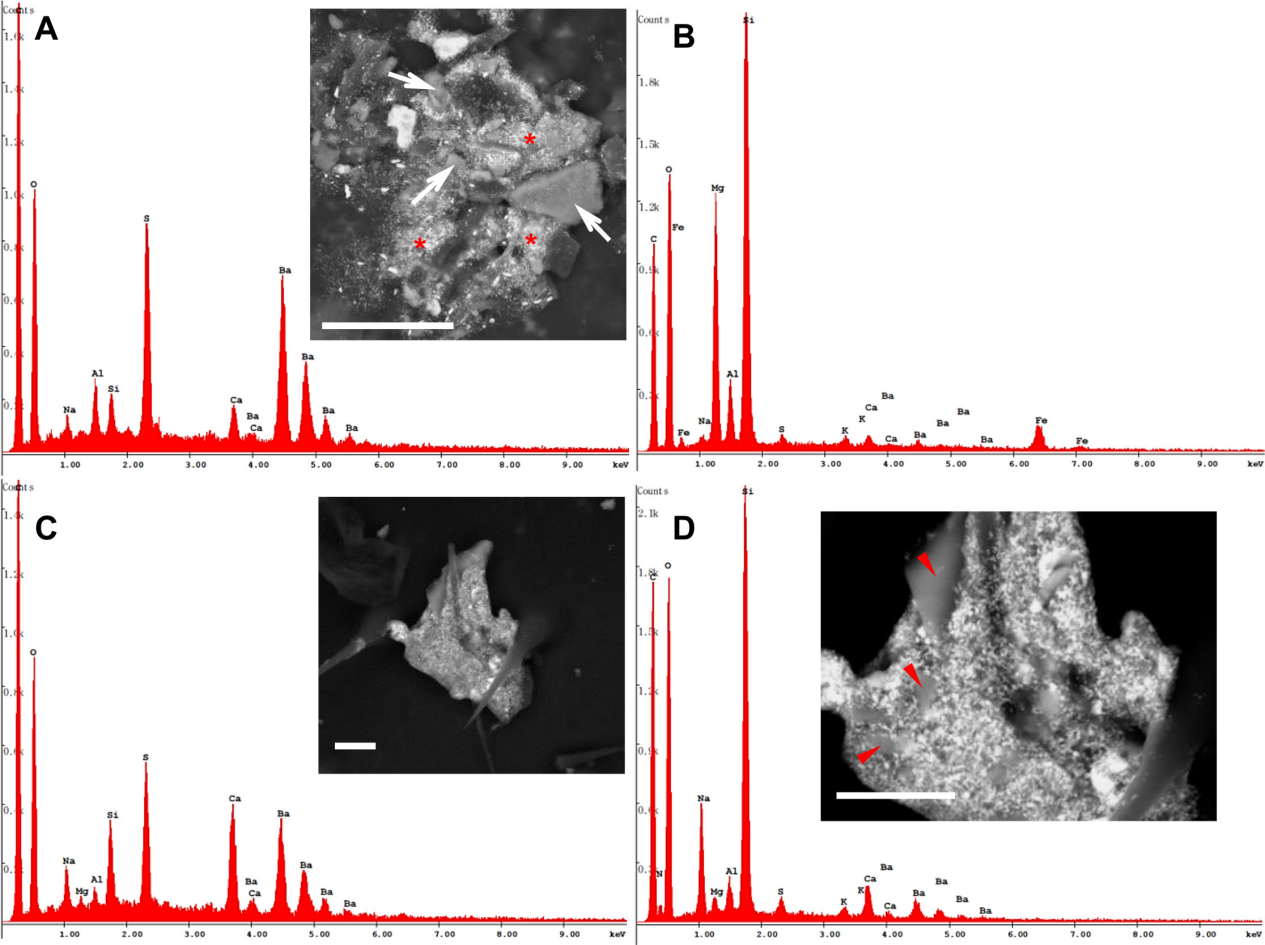
Aggregates of different mineral grains on the honey bee wings. BSE images. (A) Multi-grain aggregate of diverse mineral phases, including fine and ultra-fine grains of baryte (asterisks; EDX spectrum), and bigger particles of a phyllosilicate (arrows), whose EDX spectrum is shown in (B). Bar = 30 μm. (C) A multi-grain aggregate mainly composed of baryte. Bar = 10 μm. (D) A detail showing fragments of Na-rich plagioclase (arrowheads) and its EDX spectrum (note the contamination of baryte and possibly dolomite). Bar = 10 μm.

Finer particles were often observed adhering to and covering the bigger ones, thus forming complex, multi-grain aggregates of different mineral phases (Fig 7).

**Fig 8:**
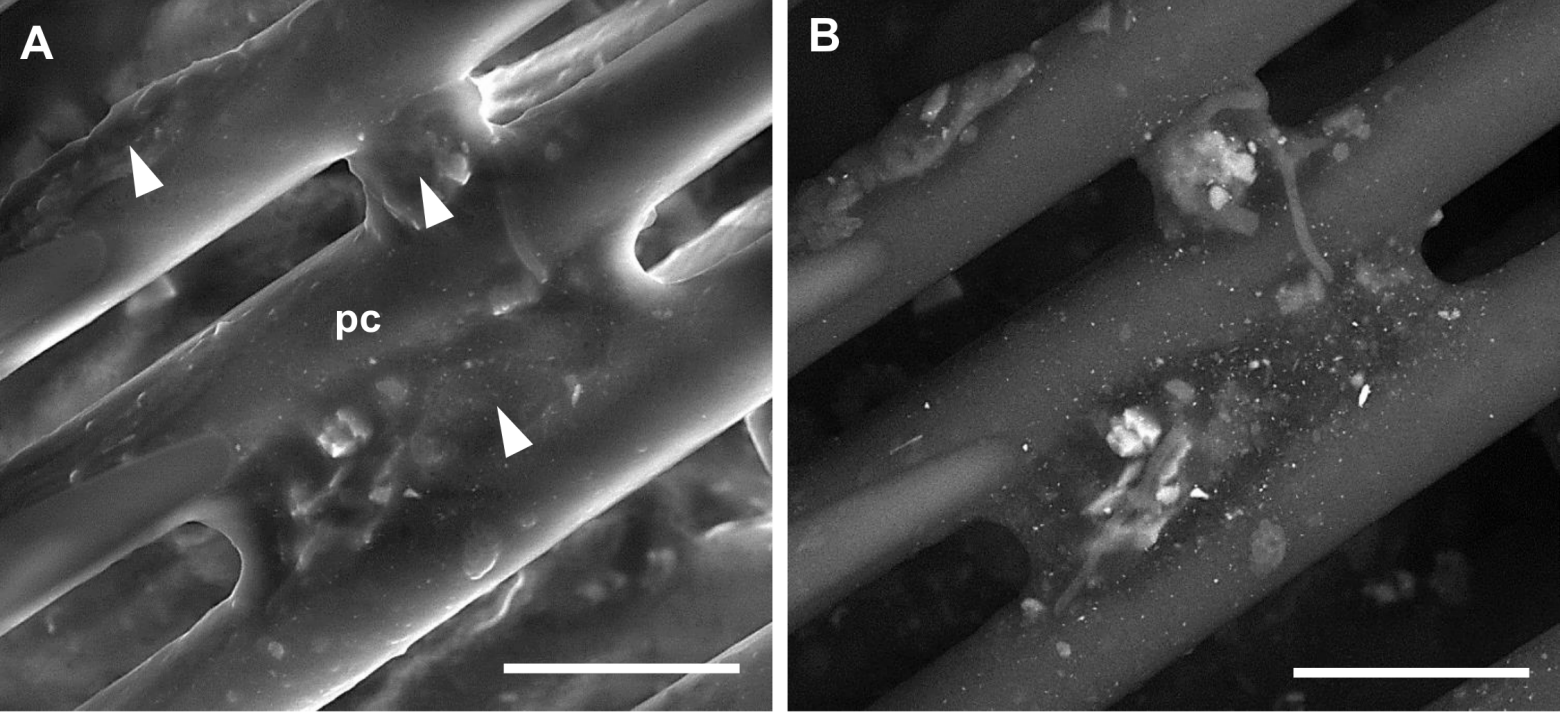
(A) SE and (B) BSE images of PM embedded in organic matrix (arrowheads) on the hind legs. pc = pollen comb. Bars = 30 μm.

Frequently, the particles were embedded in organic matrix (only C and O detected), (Fig 8).

SEM observation and X-ray spectroscopy pointed out specific morphological and chemical features of the grains. On Sardinian bees natural mineralogical phases and anthropogenic compounds have been identified (Table 1).

**Table 1:** Summary of mineral and anthropogenic compounds detected in this study.

Mineral phases	Chemical composition^a^	Honey bees	RM soil	CUN soil	BNG soil
Baryte	BaSO_4_	+*	+^c^	+	+^d^
Calcite/Aragonite	CaCO_3_	+**		+	+
Cerussite	PbCO_3_			+	
Dolomite	CaMg(CO_3_)_2_	+**		+	
Galena	PbS	+*		+	
Goethite	FeO(OH)		+^b^	+^b^	+^b^
Gypsum	CaSO_4_·nH_2_O		+^c^		
Halite	NaCl	+^§^			
Hemimorphite	Zn_4_Si_2_O_7_(OH)_2_·H_2_O		+^c^	+	
Mn oxides	MnO_2_			+	
Na-rich plagioclase	NaAlSi_3_O_8_	+**			+
Phyllosilicates	KMg_3_AlSi_3_O_10_ (OH)_2_	+**			+
Quartz	SiO_2_				+^d^
Smithsonite	ZnCO_3_		+^c^	+	
Zircon	ZrSiO_4_				+
Anthropogenic compounds containing Fe or Fe-Zn or Si-Al	n.c.^e^	+***			+

^a^ simplified after www.mindat.org, and references therein

^b^ possibly as Fe oxide coatings found on most analyzed grains

^c^ coated by Fe oxides

^d^ detected as part of multigrain aggregates

^e^ not calculated

* postmining-derived particle

** soil-derived particle

*** industry-derived particle

^§^ sea spray

**Fig 9:**
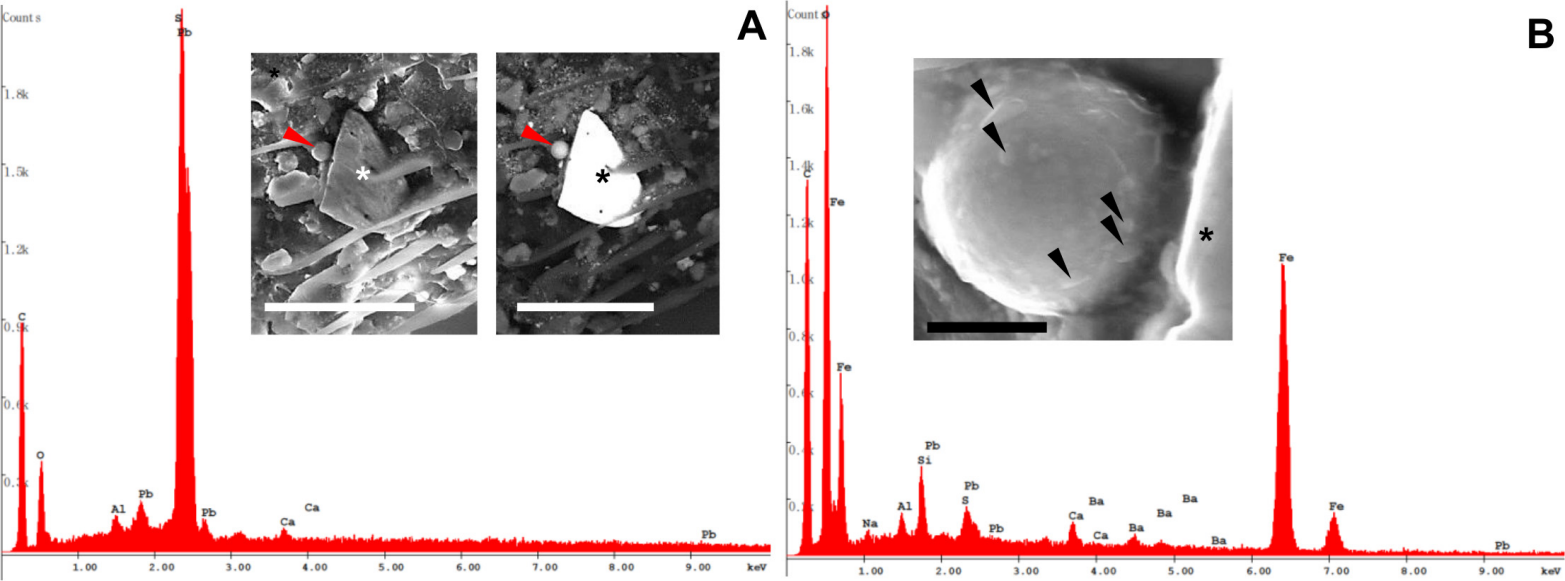
(A) A galena fragment and (B) a detail showing a Fe-rich anthropogenic particle placed on a honey bee wing. (A) SE and BSE images of a galena triangular fragment (asterisks), placed next to a rounded iron particle (arrowheads). Bars = 30 μm. (B) SE image of the rounded particle mainly constituted of iron; on its surface (partially embedded into organic matrix) are detectable nanometric grains of baryte and plagioclases (arrowheads). The Pb and S peaks are related to the galena particle (asterisk) described above. Bar = 2 μm.

Among natural phases, we were able to detect calcite/aragonite (S2A Fig) dolomite, phyllosilicates (Fig 7A, 7B and S2B Fig), and Na-rich plagioclases Fig 7D). Moreover, two mineral phases containing Ba and Pb, i.e. baryte (Figs 6 and 7) and galena (Fig 9A), were found.

**Fig 10:**
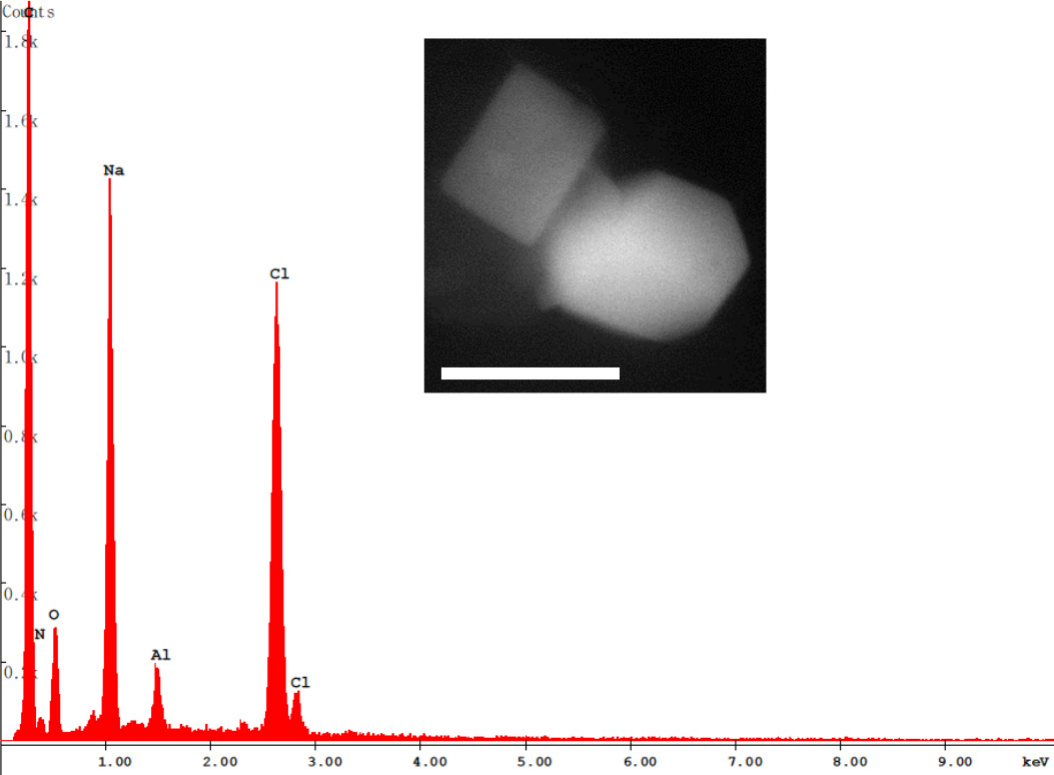
BSE image of halite found on the honey bee body. Al is possibly related to minor traces of other phases. Bars = 2 μm.

In addition, on the honey bee body, rare cubic crystals of salt (halite) were observed (Fig 10).

**Fig 11:**
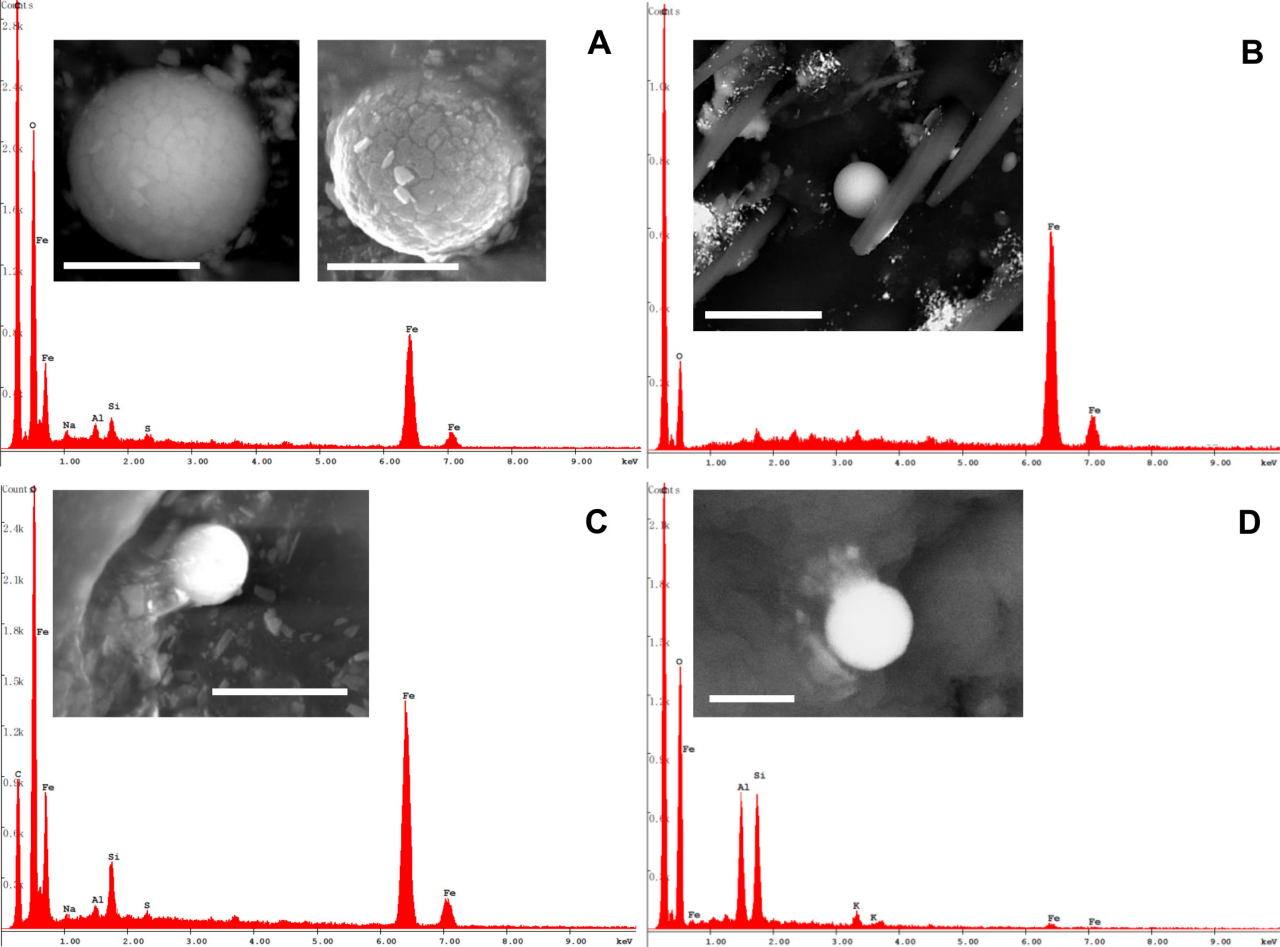
Round-shaped anthropogenic particles gathered on the honey bee wings and head surface. (A) BSE and SE images of a subspherical iron-rich particle. Note its scaly surface. Bar = 2 μm. (B) BSE image of a smooth sphere mainly constituted of iron. Several fine and ultra-fine particles of baryte (brighter fragments) are scattered all around. Bar = 10 μm. (C) SE image of rounded particle mainly constituted of iron, partially embedded into organic matrix. Bar = 2 μm. (D) BSE image of a rounded Si-Al particle, completely embedded in organic matrix (only C and O detected). Bar = 1 μm.

The honey bees also collected anthropogenic particles, which generally displayed a subspherical morphology, sometimes with a scaly surface, ranging from about 500 nm up to 10 μm in diameter (Figs 9 and 11). Characterization with EDX defined their chemistry as either Fe-rich particles or alumino-silicate (Fig 11).

**Fig 12:**
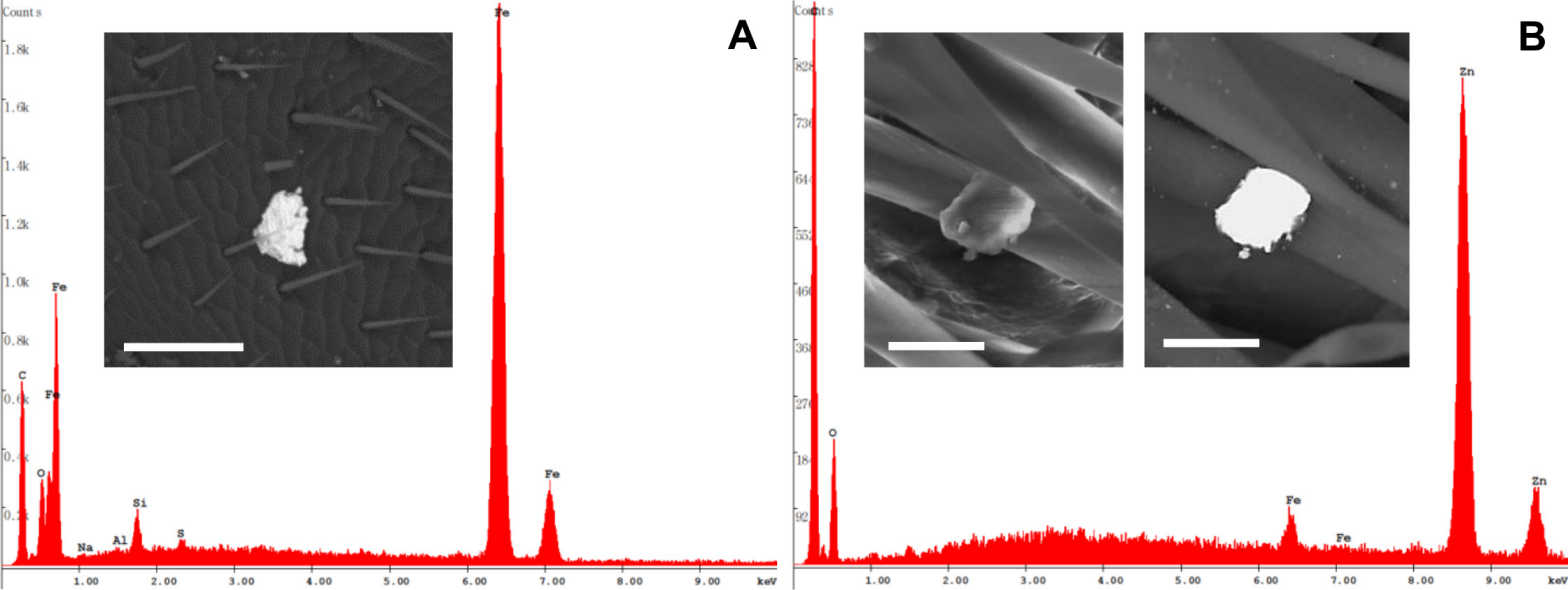
Irregular-shaped anthropogenic particles on the honey bee body. (A) Particle mainly containing Fe found on a honey bee head. Bar = 50 μm. (B) SE and BSE images showing a particle containing Zn and Fe, partially hidden by hairs of a hind leg. Bars = 10 μm.

Other anthropogenic particles showed irregular shapes and consisted of Fe or Fe combined with Zn (Fig 12).

On control bees, electronic scan detected very few PM (compared to Sardinian bees), generally located along the costal and apical margins of the fore wings (S3 Fig). Particulate, ranging from about 400 nm to 30 μm in size, was without exception composed by natural mineral phases. EDX analyses showed that singular grains (with sharp edges) or multi-grain conglomerates belonged to calcite/aragonite (S4A Fig), quartz (S4B Fig) and clay minerals.

### Honey bees: alimentary canal

**Fig 13:**
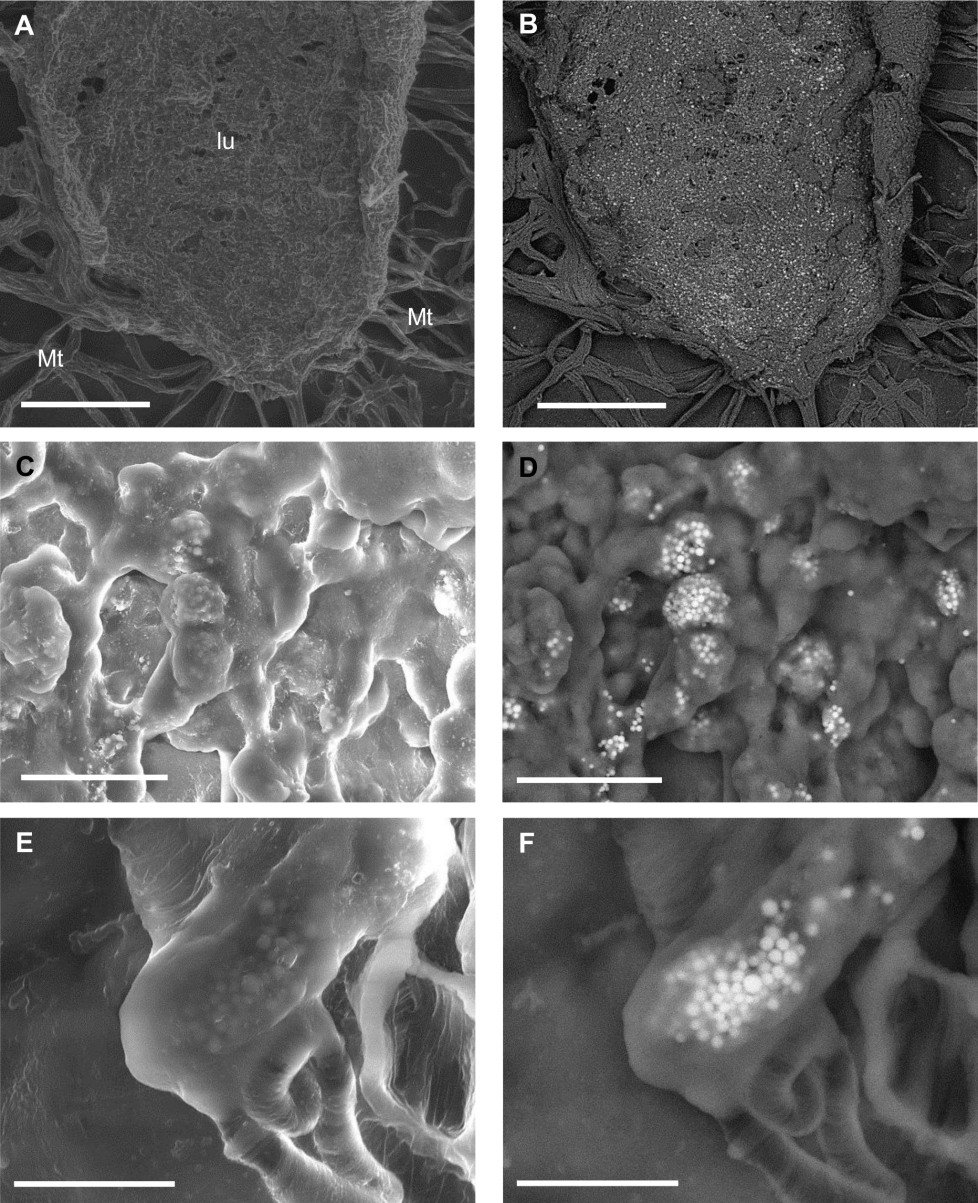
SE and BSE images of dissected portions of the alimentary canal displaying spherocrystals (brighter particles). (A, B) An opened ventriculum, showing high concentration of spherocrystals inside the epithelium. Bars = 500 μm. (C, D) A detail of the epithelium. Bars = 30 μm. (E, F) Sperocrystals in a Malpighian tubule. Bars = 10 μm. lu = lumen of the ventriculum; Mt = Malpighian tubules.

Surprisingly, the dissected alimentary canal of the hymenopterans both from Sardinia and control sites did not feature any apparent PM like that carried on the body surface. Inside the ventriculum and in the Malpighian tubules wall (close to gut) of all bees, only spherocrystals or spherites (i.e. spherical mineral concretions commonly found in many invertebrates, including insects) were detected (Fig 13).

These granules were usually grouped in grape-shaped clusters, and ranged between about 500 nm and 1.5 μm in diameter (Fig 13). EDX analyses on spherytes of both the gut and the Malpighian tubules of the Sardinian bees and control site revealed the presence of C, Ca, K, Mg, Mn, N, Na, O, P, S and Zn (S5 Fig).

### Mineralogy of sediments

Sediment analyses deliberately focused on the mineralogical composition of the wind available topsoil and top sediment cover.

The RM sites were characterized by absent soil coverage, with few exceptions (i.e. wind-repaired trenches with accumulated organic matter and litter; not sampled). The mineralogy of RM samples was distinct, with observed particle size up to 50 μm in diameter of baryte (S6A Fig), hemimorphite (S6B Fig), and smithsonite (S6C Fig). More rarely, gypsum was detected (S6D Fig). General grain habitus was subangular, reflecting cleavage and lattice structure of the individual mineral phases. Most grains were covered by a Fe-oxide layer: this attributes to the Red Muds hill a typical yellow-reddish appearance.

CUN sampling sites were located on an abandoned Pb-Zn mine dump. A portion of the dump was being colonized by shrubs and it was characterized by a thin layer of weakly, patchy developed topsoil, featuring small amounts of organic matter (not quantified). Mineral particle size was typically up to 60 μm in diameter, and the grains were frequently aggregates of smaller (<1 μm) particles. The mineralogical composition was mainly given by galena, even in tiny euhedral crystals (S7A and S7B Fig), and by fine/ultrafine grains of baryte, but it also included secondary Pb and Zn phases (e.g. cerussite, smithsonite and hemimorphite), calcite/aragonite and dolomite. Some specimens featured a partial coating by Fe- and Mn-oxides (S7B Fig).

BNG sites were the most developed, soil-wise. The sampled topsoils contained visible dark organic matter (field observation). Mineral grains were rather sporadic in this topsoil, due to relative dilution within litter and organic matter, and often appearing under the form of aggregates up to 100 μm. Main minerals detected were Na-rich plagioclase (possibly albite), calcite/aragonite, phyllosilicates and, occasionally, zircon, namely subangular to euedrally shaped (S7C Fig). Other phases, like quartz and baryte, were only observed as part of the aggregates. In BNG soil only a few subspherical anthropogenic particles were detected, and they were basically composed of Si and Al (S7D Fig).

## Discussion

### Particulate matter and its sources

During their flights and foraging activity, honey bees come into contact with different types of environmental pollutants, including airborne PM which is eventually collected on their body surface. In our study many particles resulted embedded in an organic matrix, most likely related to epicuticular waxes, i.e. the outermost layer (about 0.1–0.4 μm) of the epicuticula which envelops insect body and wings seamlessly [34]. On the legs, the organic matrix was often lifted above the surface of the body, encasing several hairs in the “sticky” matrix which may arise from the wax scales secreted by abdominal wax glands and then manipulated by the hind legs [35]. Whether entrapped in the organic matrix or simply adhering to body structures, PM is readily accessible for SEM-EDX analyses.

The honey bees used in this study did collect able to collect PM of different origin, with Sardinian insects and controls that were heavily and scarcely contaminated, respectively. On all the hymenopterans used in this study we detected mineralogical phases whose origin is linked to the natural erosion of the neighboring geological formations, i.e limestone and clay (CS), and sandstones, dolostones and shales (BNG). As soils are typically formed as a result of the weathering of the underlying bedrocks, these particles may be considered soil-derived (Table 1).

Contrary to what was observed at the control site, on BNG bees soil-derived particles were only occasionally detected, suggesting they should represent a negligible fraction in the composition of the local airborne PM.

The Sardinian hymenopterans carried a large amount of mineral phases specifically derived from past mining activity (i.e. baryte and galena), along with particles industry-derived linked to other anthropogenic activities (Table 1). Among postmining-derived PM, baryte was widespread: this is not surprising as baryte represented the main gangue component of the Pb-Zn ore deposits, containing the primary sulphides (e.g. galena) from which it was later discarded during the enrichment processes. Notably, baryte was mainly observed as fine and ultra-fine particles, whose origin can be traced back to the grinding of the barytic “tout-venant” before flotation process: this was a preparatory phase commonly held throughout the mining district of Iglesias.

At CUN, baryte has been observed in the abandoned dumps, and tiny fragments (<1 μm) were detected as part of multigrain aggregates in the sampled topsoils. In BNG samples, baryte was quite rare and always represented by fine particles adhering to larger ones to form aggregates up to 100 μm in size. Consequently, the sources of baryte found on the bees could be close to the hives. Nevertheless, we cannot exclude that the environmental contamination by this mineral involves a wider area of the “Metalliferous Ring” possibly outside the foraging range of the apiary.

In the topsoil samples from RM baryte particles were also found, but typically they were coated by iron oxides. This peculiar feature was never observed on the baryte collected by the honey bees. In addition, no other mineral phases detected in RM soils were observed on the hymenopteran body. Therefore the RM hill does not seem to be a source of PM collected by BNG honeybees.

Notably, honey bees become directly exposed to airborne particulates when they are engaged in food collection, thus for the last three weeks of their lifetime. If we also account for potential contamination by an “indoor” pollution (i.e. inside the hive), then the honey bee should not have been exposed for more than six weeks, which represents the mean lifetime of an adult worker bee. Given the considerable contamination of the bees within a very short time span, it is likely that the (human) resident population of Iglesias is liable to a non-negligible exposure to the airborne dusts detected on the hymenopterans. This can be especially the case with the fine and ultra-fine particles of baryte. Till now the exposure of the local population to nano-sized baryte fragments could have been severely underestimated. It is well known that baryte inhalation is responsible for specific respiratory pathologies such as the pneumoconiosis, a lung disease typically caused by the inhalation of mine dusts. Pirastu and colleagues have carried out epidemiological studies on the population of Sulcis-Iglesiente [24] and they demonstrated that the incidence of pneumoconiosis and other respiratory diseases (e.g. bronchitis and lung cancer), as well as renal failure, is significantly higher than the control. Moreover a causal role of atmospheric pollutants, including emissions from the metal industries and mining activities, in inducing such a kind of diseases has also been established [24].

The Sardinian bees also collected several anthropogenic compounds whose origin must be related to non-mining industrial activities (i.e. industry-derived PM).

The morphological and chemical characterization of the industry-derived particles was addressed to the identification of their potential emission sources. In particular, the subspherical iron-rich grains observed on the hymenopterans, which are the result of high-temperature combustion occurring in steelworks [10, 11], are possibly linked to the foundry/furnace dusts used at the Portovesme smelter for the Zn extraction. Specifically, both the productive processes and the inappropriate handling of the raw materials in Portovesme harbour could be the sources of this type of PM. Privately recorded videos (e.g. http://www.youtube.com/watch?v=__P6WRsp3To) confirm the spread of PM plumes in the atmosphere during loading/unloading stages. Indeed, it has already been demonstrated that the marine environment surrounding the Portoscuso-Portovesme complex (e.g. sediments and benthic foraminifera) suffers from industrial pollution [29, 36].

As for the Si–Al-rich particles, they are compatible with the atmospheric emissions of a coal-burning power plant [9–11] like the one located in the industrial settlement of Portovesme.

Whatever the emission source, pollutants from Portovesme–including its harbour where the delivery and unloading of the raw materials take place–can reach Iglesias as wind-blown particles. According to the local weather station (www.meteoiglesias.it), fall 2013 prevailing winds were mostly blowing from West–South West. Furthermore, the presence of NaCl crystals on the honey bees can be attributed to marine spray, thus confirming the preponderance of the winds blowing from the seaside.

Another candidate source of industry-derived particles is also the open pit of the Genna Luas dismissed mine, about 3 Km South of Iglesias (Fig 1A), which is a storage site for the industrial wastes from the Pb-Zn smelter of Portovesme. In the Integrated Pollution Prevention and Control (IPPC) documents (available at http://www.sardegnaambiente.it), the implementation of practices for dust abatement during the waste carrying and unloading operations in Genna Luas has been clearly indicated. Eventually, the employment of closed container trucks, water spraying systems, etc. would minimize the release of particles in the atmosphere. Nevertheless, even if Genna Luas were a source of PM, the primary source would still be attributable to the industrial settlement of Portovesme.

Finally, the anthropogenic, irregular-shaped particles essentially made of Fe or Fe combined with Zn, can be related to the process of zinc coating of iron artifacts. Indeed, in the industrial area of Iglesias a hot zinc plating plant is located, but a more detailed study is needed in order to identify the actual emission source of such peculiar particles.

### Particulate matter on the honey bee body

The present study also focused on the distribution of the PM on the bee body. Observations with the electron microscope demonstrated that particles mostly accumulated in specific body districts (Fig 2A).

The relatively clean appearance of the head, with the exception of the scape of each antenna and the narrow region close to the median ocellus, is related to the grooming behavior of the bees. Indeed, the scape falls outside the reach of the “antenna cleaner” (Fig 2A), a special grooming structure located between the first tarsal joint and the end of the tibia of the fore legs [37]. Similarly, the head midline may not be easily groomed, because cleansing movements of the fore legs are typically executed over the sides of the head, thus leaving untouched any deposited particle [30]. Considering the head as the first body part impacting the air, we have no reason to think that it would not show a relevant PM concentration without grooming activity.

The distribution of PM on the wings displayed a striking feature, as the largest amount of particles was concentrated along the costal margin and the apex of the fore wings (Fig 2A). This can be explained by the peculiar aerodynamic of the insect flight in which, during each stroke, airflow separates at the edge of the wings, forming a stably swirling structure called Leading Edge Vortex (Fig 2B) [38–40]. As a consequence, it is possible that the airborne dusts are continuously entrapped and canalized by the air flow, eventually becoming in contact with the wing edge where they adhere to the epicuticular wax and the hairs.

On the hind legs PM was gathered mainly on structures involved in the body grooming, pollen collection and wax handling (i.e. the pecten on the lower end of the tibia, and the pollen comb on the metatarsus). Since at the time of sampling the bees were exclusively collecting honeydew on holm oaks (no flowering was in place both at BNG and control site), only rare pollen grains were observed on the body surface. Nonetheless, even in absence of pollen, grooming behavior continues, allowing airborne dust to be collected and transferred from the first and second pairs of legs to the hind legs [30], where at last PM accumulates.

Interestingly, inside the alimentary canal no trace of PM was found. Airborne particles can be swallowed by worker bees throughout their foraging activity. During our sampling, bees were exclusively collecting honeydew, as confirmed by the absence of pollen grains inside the dissected alimentary canal. While it has been demonstrated that anthropogenic dust can adhere on the pollen surface [41] and a subsequent ingestion of particulate by honey bees feeding on contaminated pollens cannot be ruled out, the presence of PM in the honeydew may be questionable. In fact, a contamination of the honeydew by passive deposition of airborne particles (fallout) is unlikely, because bees are known to always prefer freshly secreted honeydew droplets to older ones [42]. Analogously, a direct contamination of the honeydew is unlikely because this would assume that solid PM would enter through the stomata of the oak leaf surface, sequentially reaching the plant phloem, the honeydew secreted by the sap-feeding insects and, eventually, the honey bee.

Since additional transport mechanisms of particulate do exist (e.g. water ingestion and/or air inhalation via spiracles), further analyses involving other organs and tissues of the bees must be carried out in order to definitely exclude the intra-body presence of PM.

The ventriculum and Malpighian tubules of the bees from both Sardinian and control sites showed a conspicuous number of spherocrystals (spherites), as commonly found in many insect species, where it has been demonstrated that they originates from the endoplasmic reticulum–Golgi complex [43, 44]. The function of these granules, mainly composed by phosphates and/or urates, may be related to ionic and homeostatic regulation, but they also seem to serve as storage sites of essential inorganic compounds, and even toxic waste materials as heavy metals [44, 45].

Interestingly, the honey bees from both the Sardinian polluted area and the control site carried spherites characterized by the same chemical elements (C, Ca, K, Mg, Mn, N, Na, O, P, S, Zn), as already reported in other insect species [43–45].

In the surroundings of Iglesias, heavy metals such as Pb, Fe and Ba were environmentally available and also detected in the PM adhering to the hymenopteran body surface. The absence of such elements from the spherocrystals found in BNG bees suggests that the elemental composition of spherites does not strictly depend upon environmental context. Thus it is likely that the primary function of these concretions is not linked to a specific mineral detoxification of the internal milieu.

Additional investigations are needed in order to clearly define the exact role of the spherocrystals in the honey bees, and eventually to trace the fate of potential toxic elements redistributed via PM in Sulcis-Iglesiente.

## Conclusions

Because of its mining and industrial history, Sulcis-Iglesiente is a highly polluted region where heavy metals and metalloids can quickly access the food chain. Usually water contamination is considered the key source for elemental mobility and this leads to overlook other diffusional routes of toxic elements, such as airborne dust (i.e. solid state pollutants).

In this study we showed that honey bees, reared in the Iglesias municipality, collected airborne PM on their own body in a form easily accessible to the morpho-chemical analyses. In no more than three weeks, the body of the worker bees was covered by thousands of particles, mainly derived from the past mining activities, but also from the industrial processes taking place in the neighboring areas. Consequently, the exposure of the local human population to airborne PM may have been severely underrated and underestimated.

On the whole, the present study aims to become the starting point for a new and promising avenue of research in the field of environmental monitoring. We are confident that the contribution of the honey bees as “living samplers” of airborne dust will be crucial in assessing the air quality in a broad variety of contaminated environment, by adding valuable contributions to the data provided by fixed air sampling systems commonly employed for monitoring airborne PM.

## Supporting Information

S1 Fig BSE image of a hind wing with scattered PM (bright spots). Bar = 1 mm.
(TIF)

S2 Fig Soil-derived particles detected on the honey bee body. (A) Calcite/aragonite (Bar = 30 μm) and (B) phyllosylicate (Bar = 10 μm).
(TIF)

S3 Fig BSE image of a hind wing of a control bee. Bar = 1 mm. (TIF)

S4 Fig (A) Calcite/aragonite (Bar = 20 μm) and (B) quartz (Bar = 50 μm), detected on the control bees. (TIF)

S5 Fig EDX spectrum showing the chemical composition of the spherocrystals in the Sardinian bees. (TIF)

S6 Fig (A) Baryte (Bar = 3 μm), (B) hemimorphite (Bar = 3 μm), (C) smithsonite (Bar = 5 μm), and (D) gypsum (Bar = 5 μm) detected in the Red Muds soil samples. (TIF)

S7 Fig (A) Euhedral crystal of galena partially covered by Fe-oxides, hemimorphite and dolomite grains (Bar = 3 μm). (B) Galena coated by Fe and Mn-oxides, hemimorphite and dolomite grains (Bar = 3 μm). Mine dump of Cungiaus. (C) Zircon (Bar = 30 μm) and a subspherical industry-derived particle of Si-Al (Bar = 30 μm), detected in Bingiargia soil samples.
(TIF)
