# Supplementary figures and images for: Honey Bees (Apis mellifera, L.) as Active Samplers of Airborne Particulate Matter
Source: PLoS One. 2015 Jul 6;10(7):e0132491. doi: 10.1371/journal.pone.0132491 (PMC4492680; doi:10.1371/journal.pone.0132491)

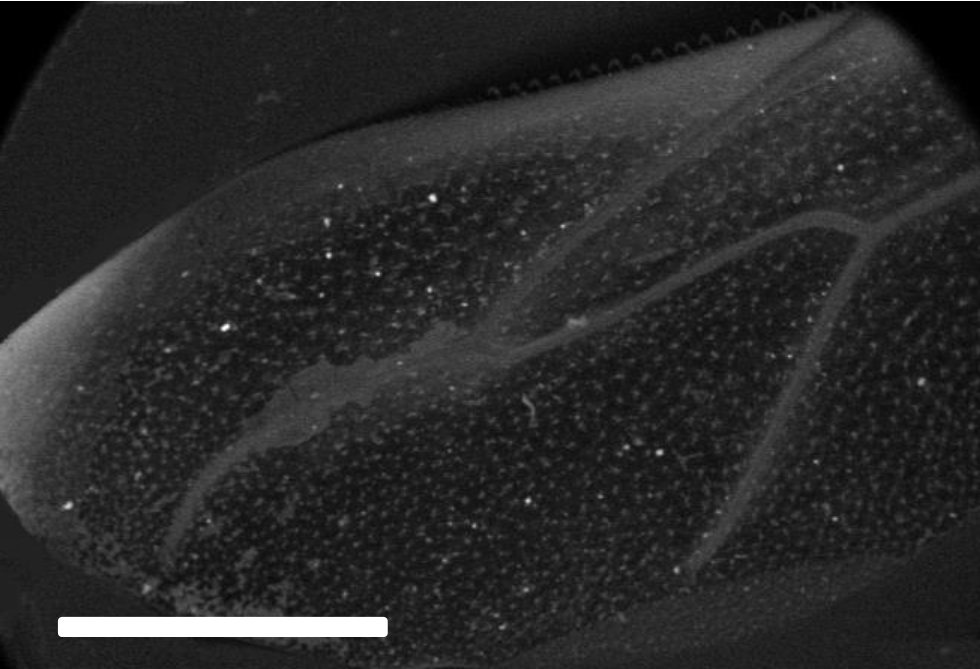

Supplement: S1 Fig — Bar = 1 mm. (TIF) [file pone.0132491.s001.tif]

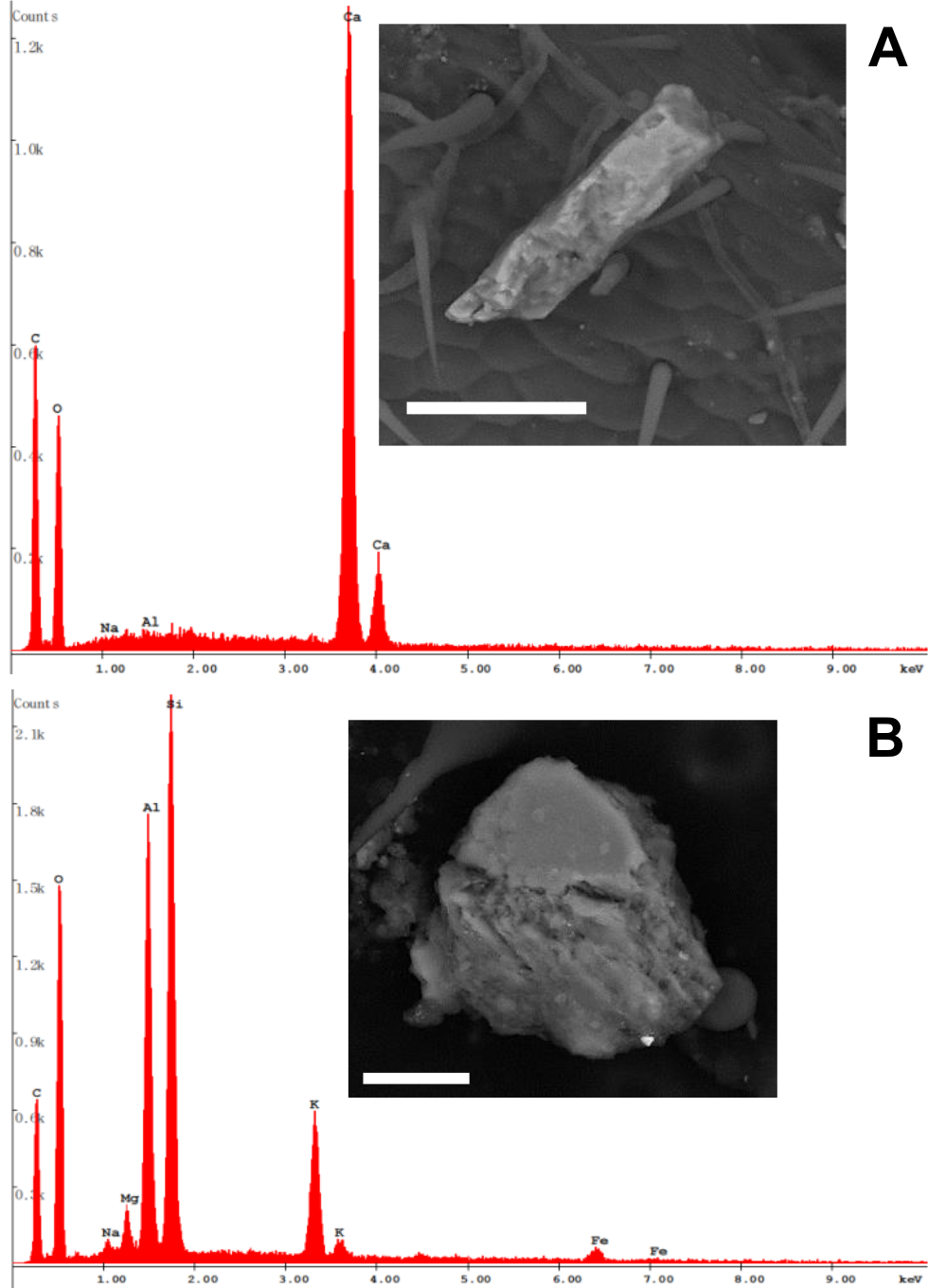

Supplement: S2 Fig — (A) Calcite/aragonite (Bar = 30 μm) and (B) phyllosylicate (Bar = 10 μm). (TIF) [file pone.0132491.s002.tif]

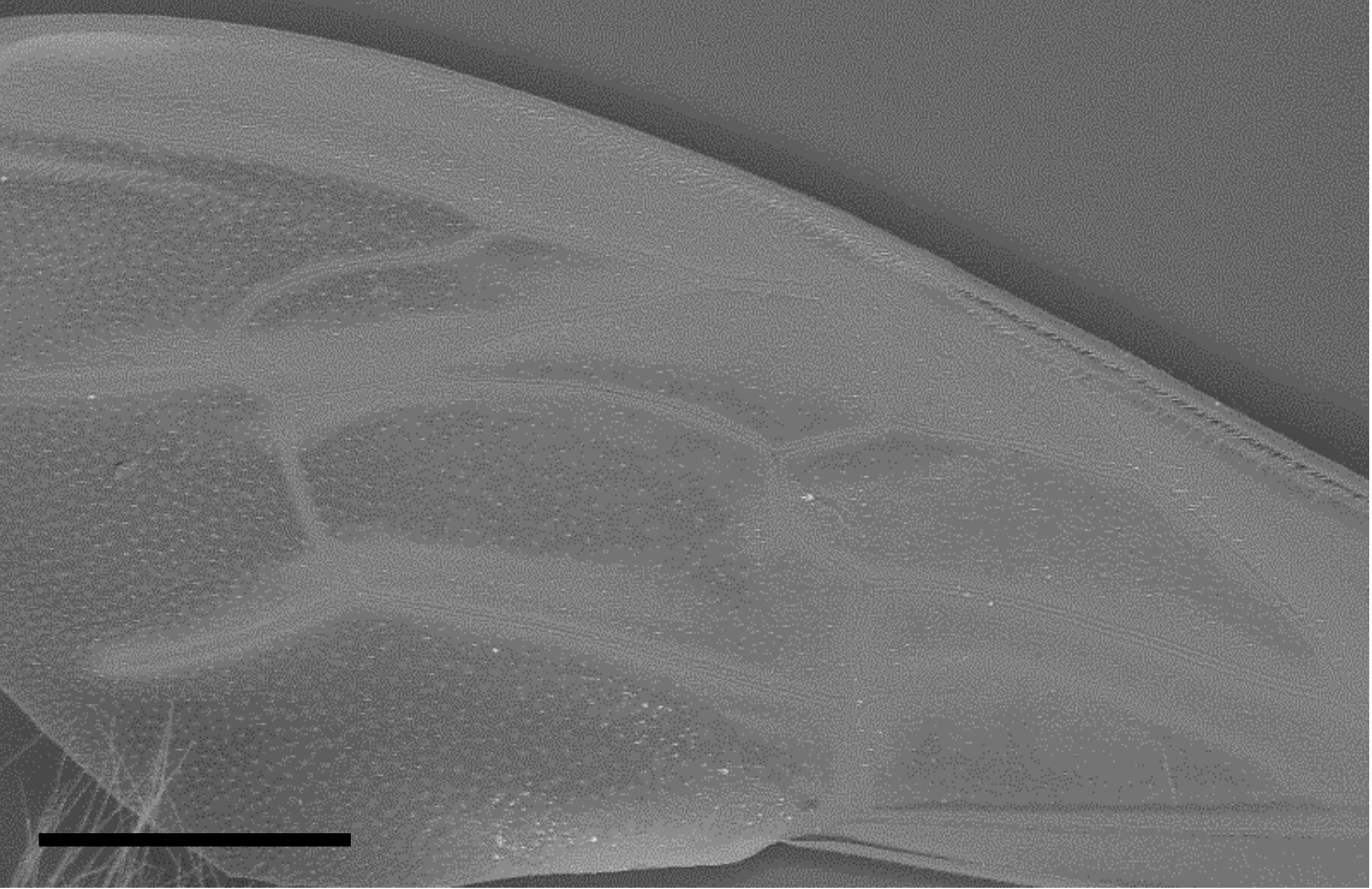

Supplement: S3 Fig — (TIF) [file pone.0132491.s003.tif]

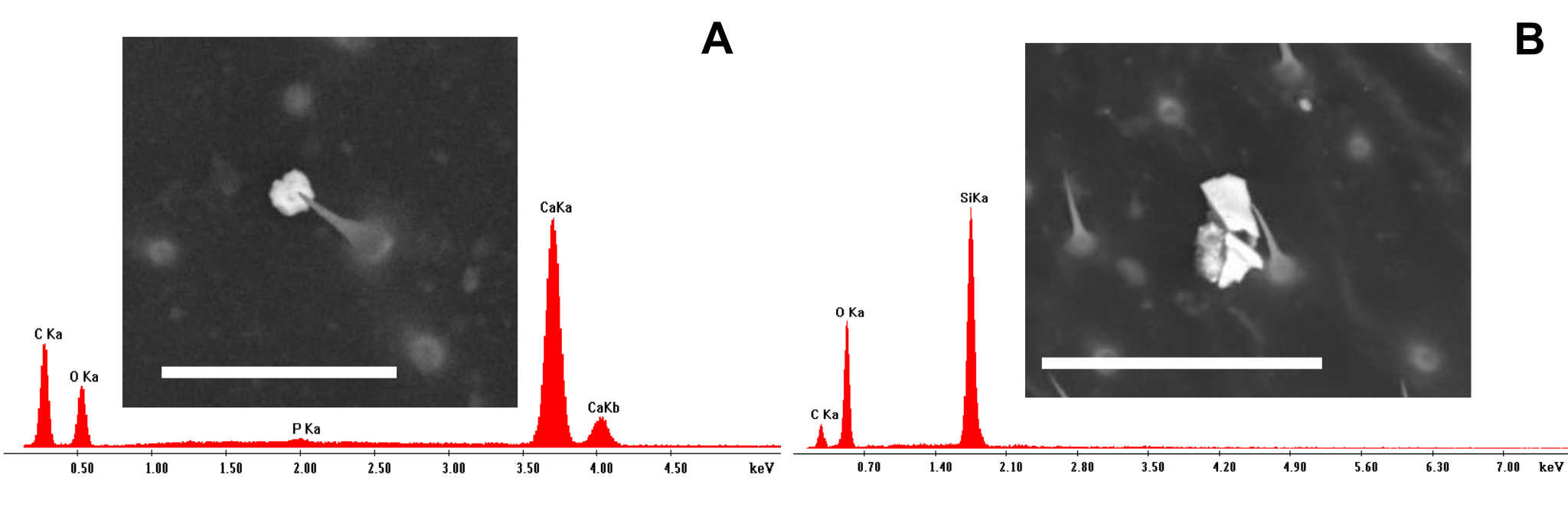

Supplement: S4 Fig — (TIF) [file pone.0132491.s004.tif]

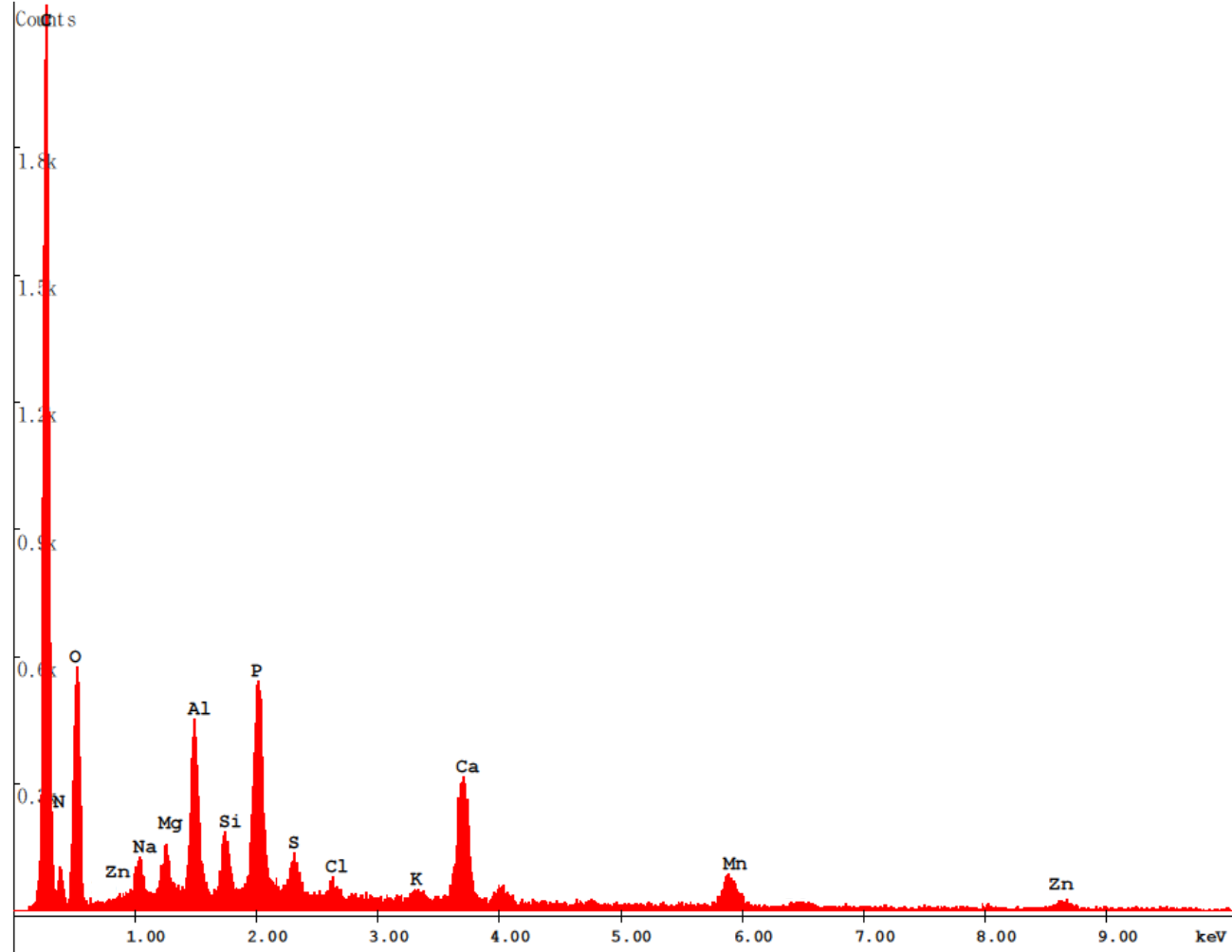

Supplement: S5 Fig — (TIF) [file pone.0132491.s005.tif]

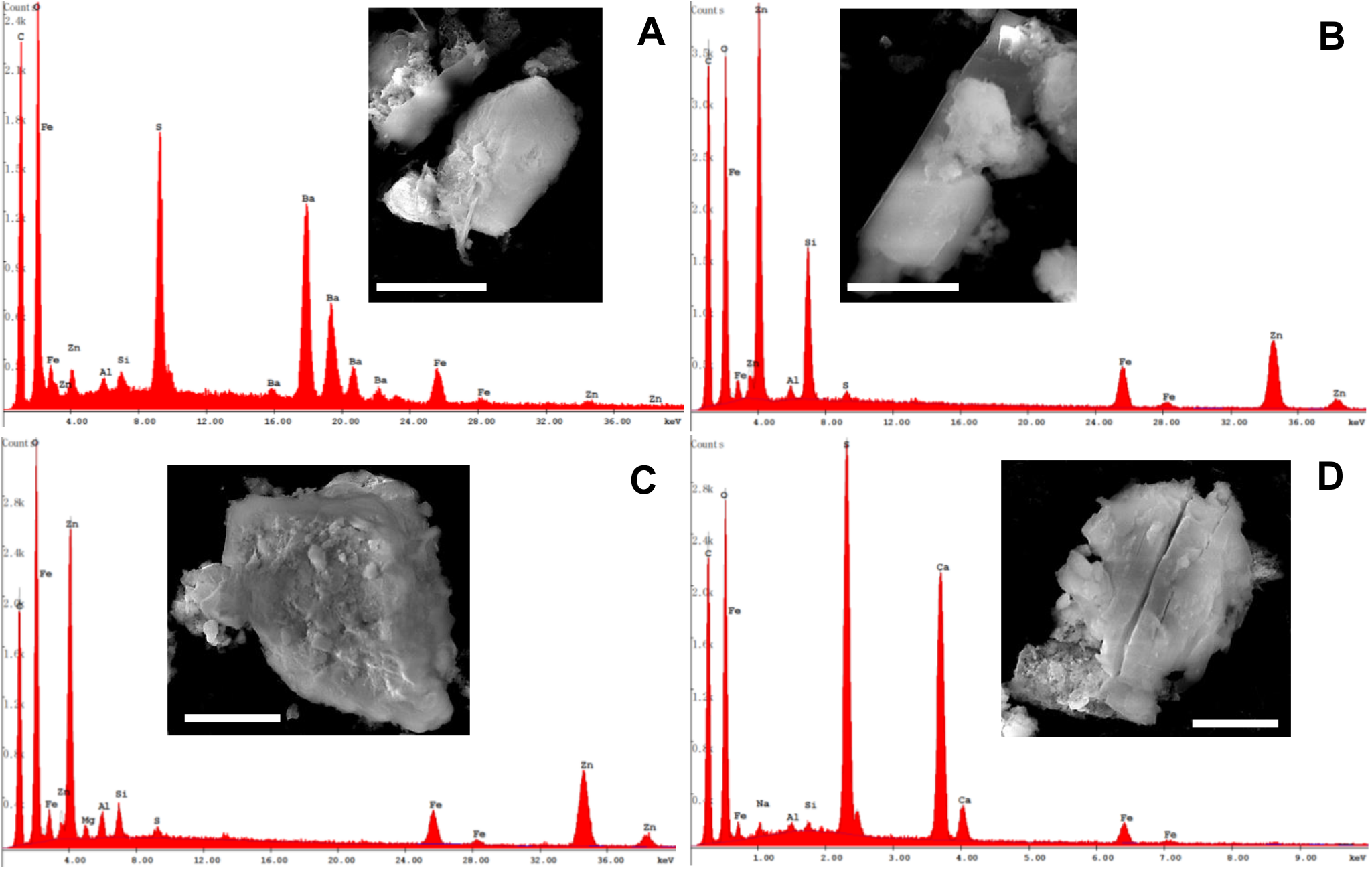

Supplement: S6 Fig — (TIF) [file pone.0132491.s006.tif]

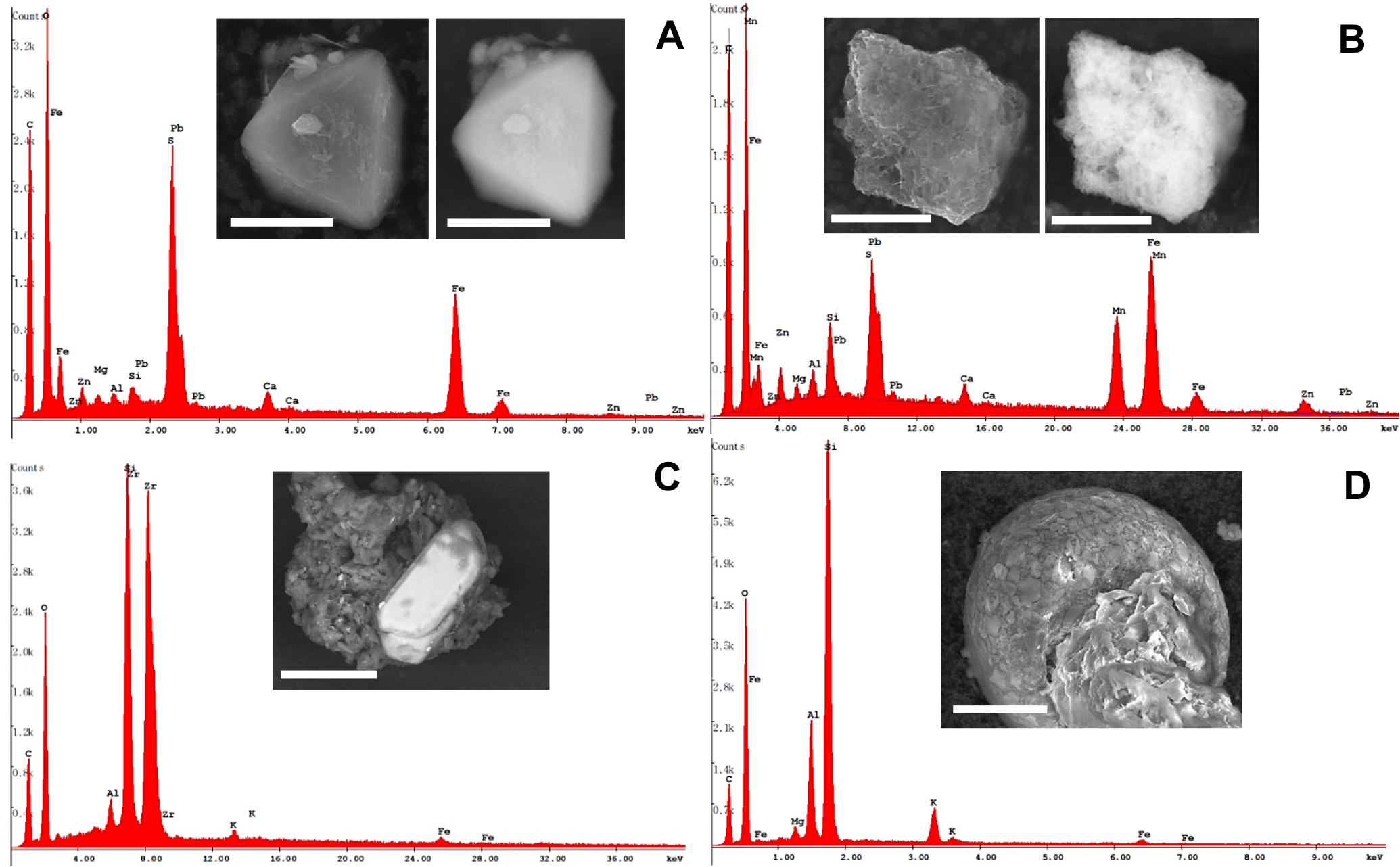

Supplement: S7 Fig — (B) Galena coated by Fe and Mn-oxides, hemimorphite and dolomite grains (Bar = 3 μm). Mine dump of Cungiaus. (C) Zircon (Bar = 30 μm) and a subspherical industry-derived particle of Si-Al (Bar = 30 μm), detected in Bingiargia soil samples. (TIF) [file pone.0132491.s007.tif]
